# Supplementary material for: Multiplexed quantitative proteomics provides mechanistic cues for malaria severity and complexity
Source: Commun Biol. 2020 Nov 17;3:683. doi: 10.1038/s42003-020-01384-4 (PMC7672109; doi:10.1038/s42003-020-01384-4)
Supplement: Supplementary file 1 — Supplementary Information [file 42003_2020_1384_MOESM1_ESM.pdf]

Supplementary Materials

Supplementary Table 1. List of significantly altered common host proteins in SFM and NSFM as compared to healthy control from plasma samples.

SFM vs. HC	Common proteins	NSFM vs. HC
P02760	V9GYM3	O60476
Q08380	P02750	P25311
A0A0B4J231	A0A0A0MTS7	P02741
P61769	P02763	P02671
P05362	B4E1Z4	P02774
P02790	P00738	P06681
P19320	P02746	A0A0G2JMY9
Q15699	H0YCJ8	P04196
P16070	P11226	P19652
A0A0A0MS08	P0DOY2	Q14624
O75636	P02675	P04004
P01008	P05090	Q04756
P02647	P01614	P08519
Q06033	P04040	P27169
P00734	P68871	P04217
Q9NZP8	P02649	
P00739		
P02787		
A0A286YEY4		
P02747		
P06727		
P01834		

Supplementary Table 2. List of significantly altered host proteins in SA and CB patient plasma when compared to healthy control.

CB vs. HC	Common proteins	SA vs. HC
P02746	V9GYM3	Q04756
P02741	H0YCJ8	P27169
P68871	P04040	A0A0A0MTS7
Q15699	P02647	P01034
P02790	P02775	P0DOY2
P02649	P02750	P11226
P05362	P00738	P07358
P08519	P02763	P60709
P49908	B4E1Z4	P17936
P0DJI8	P02787	P04180
P07357	P02776	P02654
P06727	P00734	P01024
P01011	P02774	A0A286YES1
C9JV77	A0A0G2JMY9	
B4DPQ0	P61769	
Q06033		
P19652		
P10643		
O60476		
Q8NF91		

Supplementary Table 3. List of significantly altered host proteins in NSFM and NSVM patient plasma when compared to healthy control.

NSFM vs. HC	Common proteins	NSVM vs. HC
A0A0A0MTS7	P02671	P19827
P00738	P02750	P02647
H0YCJ8	P02741	P02775
Q14624	P02763	O95445
O60476	B4E1Z4	C9JF17
P25311	P02675	P00450
P02774	V9GYM3	P02654
P02746	P04196	P19823
P06681		P06396
A0A0G2JMY9		P04264
P11226		P07360
P19652		P09871
P04004		P01011
Q04756		J3KPS3
P0DOY2		P02748
P05090		
P01614		
P08519		
P04040		
P68871		
P27169		
P02649		
P04217		

Supplementary Table 4. Clinicopathological analysis of hematological and biochemical parameters in healthy controls and the patients suffering from NSFM, SFM_Others, CB, SA, VM and DF.

	Healthy control (HC = 58)	Non-severe Falciparum Malaria (NSFM, n = 15)	Severe Falciparum Malaria (SFM, n = 38)*	Other complications (SFM_others, n = 27)#	Cerebral Malaria (CB, n = 7)	Severe Anemia (SA, n = 5)ϕ	Falciparum malaria (FM, n = 53)∸	Vivax malaria (VM, n = 16)≈	Dengue Fever (DF, n = 15)
**Hematological parameters**									
**Hemoglobin (g/dl)**	13.3 (11.8-14.7)	12 (5-14)	10.5 (3.7-15.9)	11 (6.1-15.9)	11.7 (7.5-13)	4.25 (3.7-4.9)	11.2 (3.7-15.9)	10.29 (6.8-13.9)	13.55 (9.9-17.8)
**Platelets**	3 (1 - 4.3)	1.7 (0.46-4)	0.75 (0.19-3.1)	0.895 (0.19-3.1)	1.3 (0.3-2.9)	0.66 (0.2-2.05)	1.05 (0.19-4)	2.3 (0.8-4.6)	0.56 (0.1-1.3)
**Biochemical parameters**									
**SGOT**	36 (23 -74)	29 (23-110)	62 (11-340)	62 (12-340)	37 (11-210)	68 (24-234)	36 (23-74)	54 (11-340)	45 (14-140)
**SGPT**	38 (19 -87)	35 (15-114)	81 (15-459)	81 (15-243)	92 (32-300)	57 (17-459)	38 (19-87)	63 (15-459)	41 (18-92)
**ALP**	104 (66-145)	108 (66-289)	157 (12-427)	159 (43-427)	157 (13-254)	123 (12-188)	104 (66-145)	145 (12-427)	93 (42-256)
**Total Bilirubin**	0.9 (0.3-2)	1.2 (0.6-2.9)	1.9 (0.45-11.96)	1.6 (0.45-7.2)	1.9 (0.7-5.7)	1.35 (1.1-2.9)	0.9 (0.3-2)	1.4 (0.45-7.2)	0.9 (0.9-2.1)

Data represented as mean (lowest value – highest value).
#SFM_others, n is variable for different parameters: SGPT and ALP (n = 25), and Total Bilirubin (n = 23).
ϕ SA, n is variable for different parameters: Total Bilirubin (n = 4).
∸FM, n is variable for different parameters: SGPT (n = 52), and Total Bilirubin (n = 49).
≈VM, n is variable for different parameters: Platelets, SGPT and ALP (n = 17), and Total Bilirubin (n = 4).

Supplementary Table 5. PCR mix composition for nested PCR

S.no.	Components	Volume	Working concentration	Stock concentration
**1**	Buffer	2.5 µl	1X	10X
**2**	dNTP	2 µl	0.2 mM	2mM
**3**	Primer F.g	0.8 µl	0.5 µM	2mM
**4**	Primer R.g	0.8 µl	0.5 µM	2mM
**5**	DNA template	-		
**6**	DNA polymerase	0.25 µl	1.0 units/50 µl	
**7**	Double distilled water	18.65 µl		
	Total	25 µl		

Supplementary Table 6. PCR run parameters

S.no.	Step	Temperature - Time
**1**	Prolong denaturation	94 ^{0}C – 4 min
**2**	Denaturation	94 ^{0}C – 40 sec
**3**	Annealing	58 ^{0}C – 1 min
**4**	Extension	68 ^{0}C – 2 min
**5**	Go to step 2	30 repeats
**6**	Prolong extension	68 ^{0}C – 5 min

Supplementary Table 7. WHO case definition of severe malaria. The infection is considered as severe if the patients exhibit one or more of these following conditions.

S.no.	Complication	Condition criteria for adults
1.	Impaired consciousness	A Glasgow Coma Score <11
2.	Acidosis<br>Clinical manifestation respiratory distress	>8 meq/l Base deficit or, <15 mM bicarbonate in plasma or >5 mM plasma lactate.
3.	Hypoglycemia	Glucose level in blood or plasma <2.2 mM (<40 mg/dl)
4.	Severe malarial anemia	<7 g/dl of haemoglobin concentration or <20% of hematocrit together with a parasite count >10 000/µl
5.	Renal impairment	>265 µM (3 mg/dl) of creatinine in Plasma or serum or >20 mM urea in blood
6.	Jaundice	>50 µM (3 mg/dl) of bilirubin in plasma or serum with >100 000/µl parasite count
7.	Pulmonary edema	Radiologically confirmed, or >30/min respiratory rate with <92% oxygen saturation in air
8.	Significant bleeding	Recurrent or prolonged bleeding from nose gums or venipuncture sites; hematemesis or melena
9.	Compensated Shock	≥3 s capillary refill or temperature gradient on leg (mid to proximal limb), but no hypotension. Decompensated shock is defined as systolic blood pressure <70 mm Hg in children or <80 mm Hg in adults with evidence of impaired perfusion (cool peripheries or prolonged capillary refill)
10.	*Hyper-parasitemia	>10% *P. falciparum* parasitemia

* Hyper-parasitemia is not applicable in defining *P. vivax* severity as the pathogen can cause severe infections even at a low level of parasitemia.

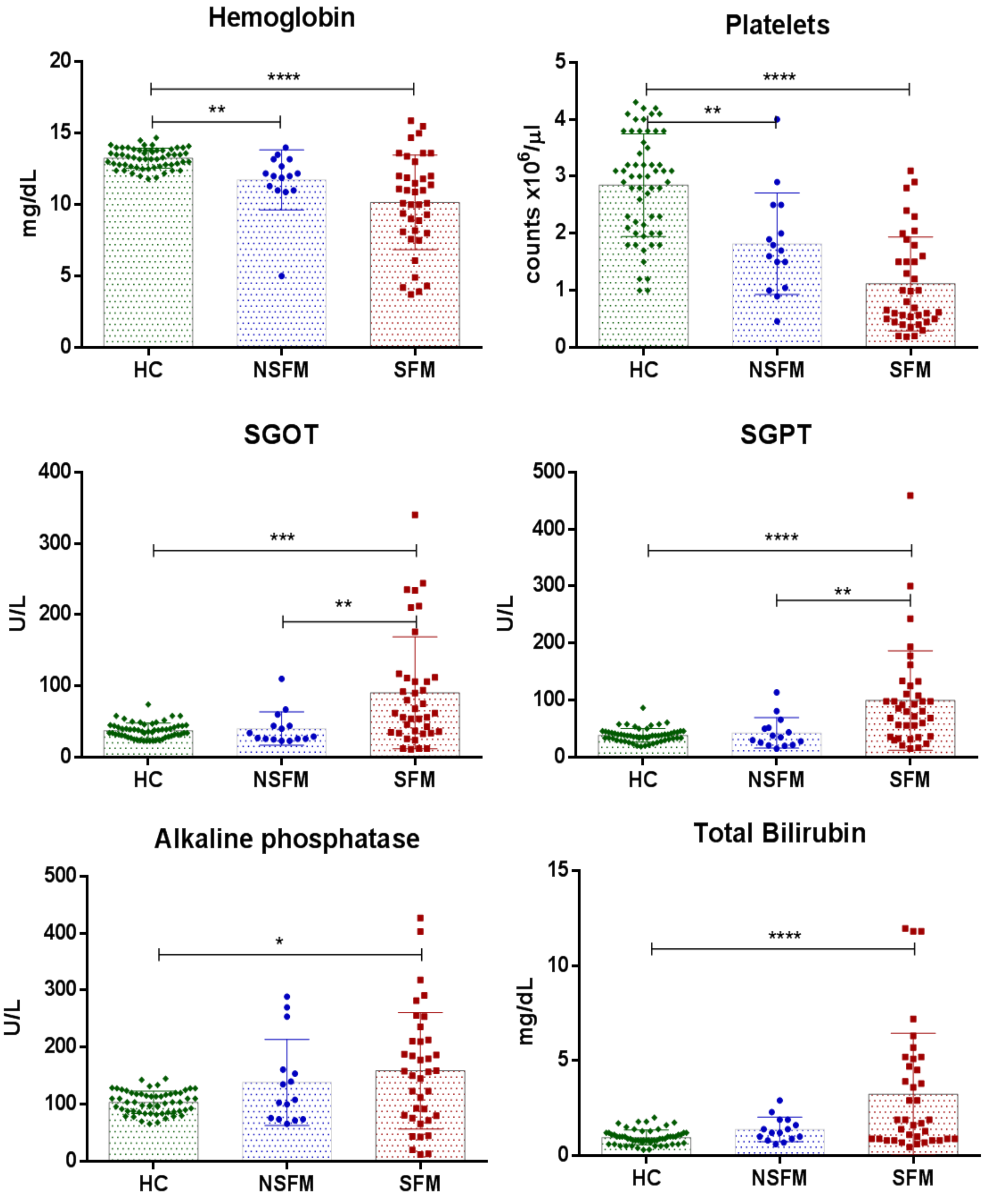

Supplementary Fig. 1 Analysis of clinical parameters in severe falciparum malaria (SFM; n = 26), non-severe falciparum (NSFM; n = 15) and healthy control (HC; n = 58). All data represented as median ± SE, (*, $p \leq 0.05$), (**, $p \leq 0.01$), (***, $p \leq 0.001$), (****, $p \leq 0.0001$) in student's t-test.

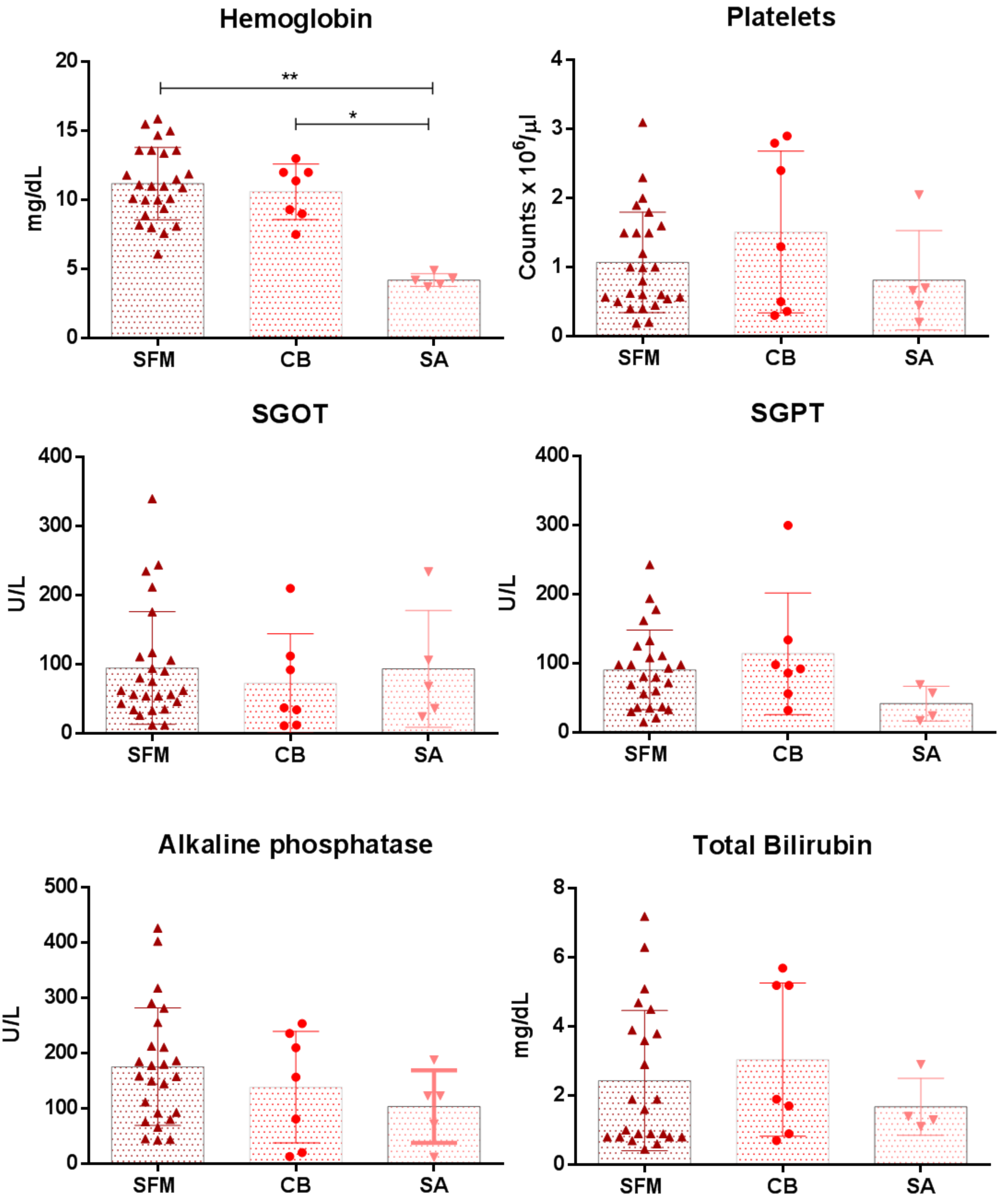

Supplementary Fig. 2 Analysis of clinical parameters of severe falciparum malaria (SFM; n = 26) patients (#SFM: severe falciparum malaria except for cerebral and severe anemia), cerebral malaria (CB; n = 7) and severe anemia (SA; n = 5). All data represented as median ± SE, (*, $p \leq 0.05$), (**, $p \leq 0.01$) in student's t-test.

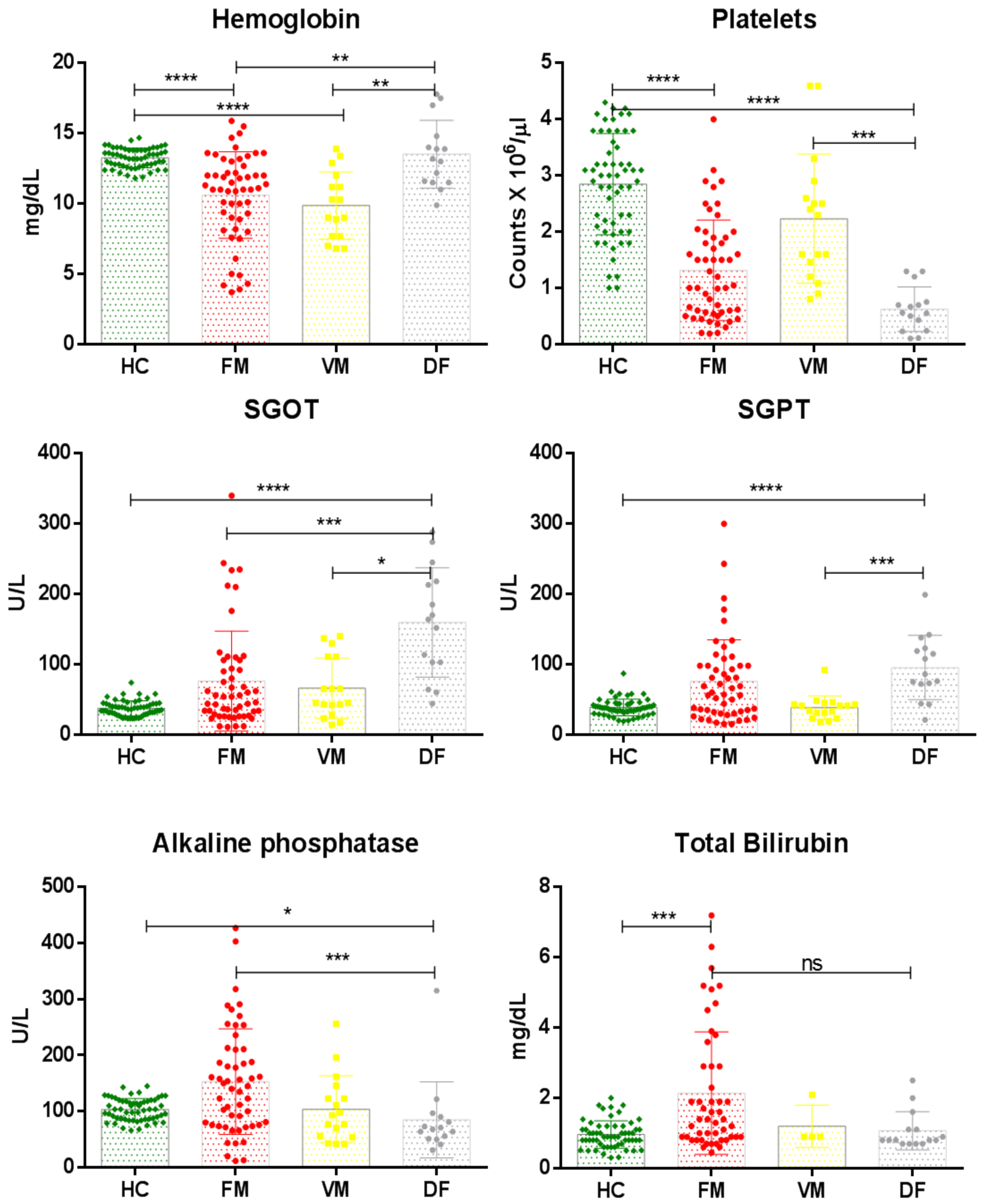

Supplementary Fig. 3 Analysis of clinical parameters in falciparum malaria (FM; n = 53, it includes non-severe and severe cases of falciparum malaria), vivax malaria (VM; n = 17, it includes non-severe and severe cases of vivax malaria), dengue fever (DF; n = 15, It includes non-severe and severe dengue) and Healthy control (HC). All data represented as median ± SE, (*, $p \leq 0.05$), (**, $p \leq 0.01$), (***, $p \leq 0.001$), (****, $p \leq 0.0001$) in student's t-test.

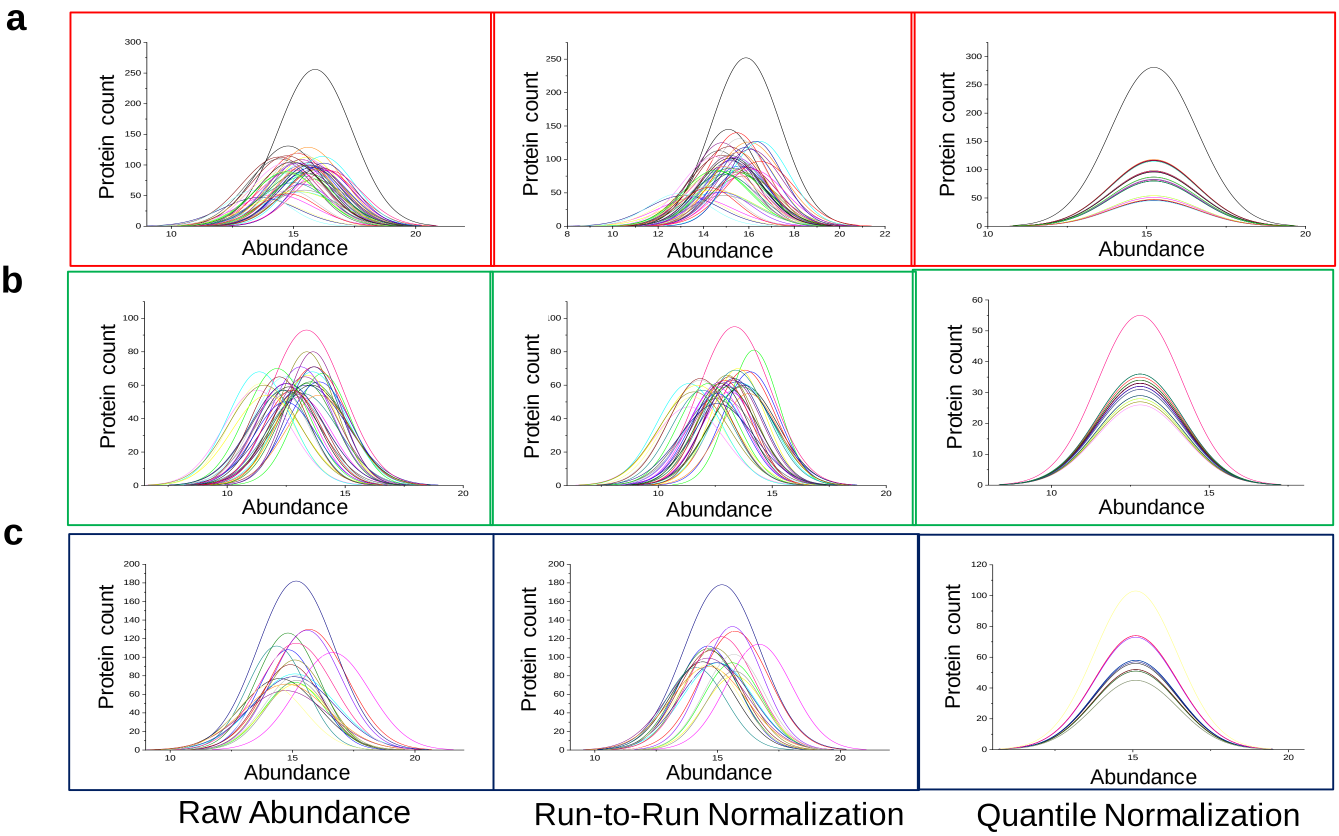

Supplementary Fig. 4 Quality control check and pre-processing of proteomic data. **a-c** Density plots representing the proteome expression (TMT-based quantification) profiles of Falciparum malaria **a**, Vivax malaria **b,** and Dengue **c,** data sets. We plotted the protein abundance values from raw data, which were sequentially normalized by reference pool and quantile normalization. There profile density plots are representing the distribution of abundance of all proteins in one plot. Y axis shows the no. of proteins count and x-axis shows the abundance of the proteins in log2 values. We observed that the raw data were not perfectly normally distributed, while after the normalization, the data are normally distributed (symmetrical distribution indicating that the normalization methods effectively reduced the variations observed in the raw data sets).

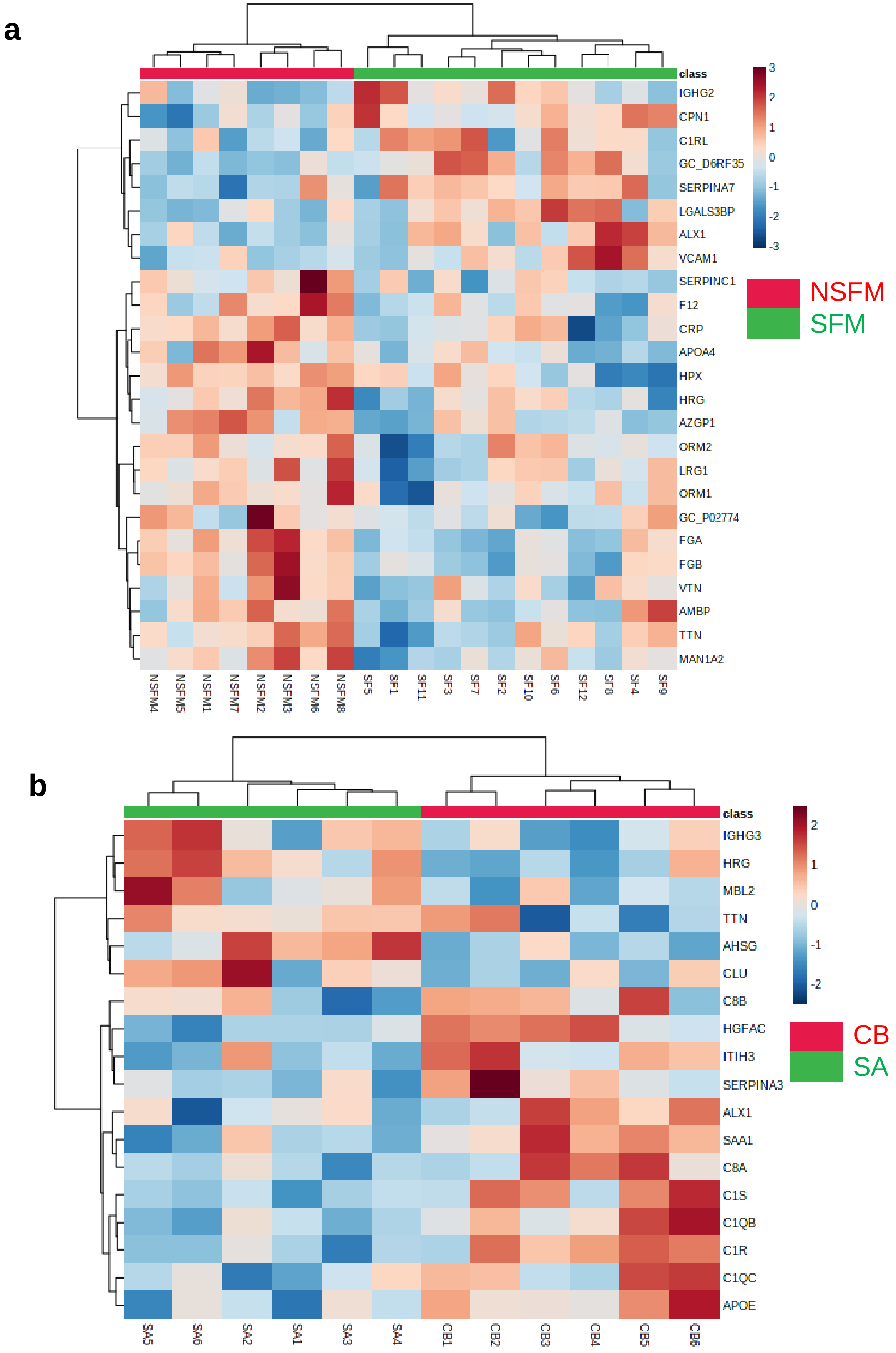

Supplementary Fig. 5 Unsupervised clustering of significantly altered proteins ($p < 0.05$) in falciparum malaria. **a** Heat map of individual samples for non-severe falciparum malaria (NSFM) and severe falciparum malaria (SFM). **b** Heat map of individual samples for cerebral malaria (CB) and severe malaria anemia (SA).

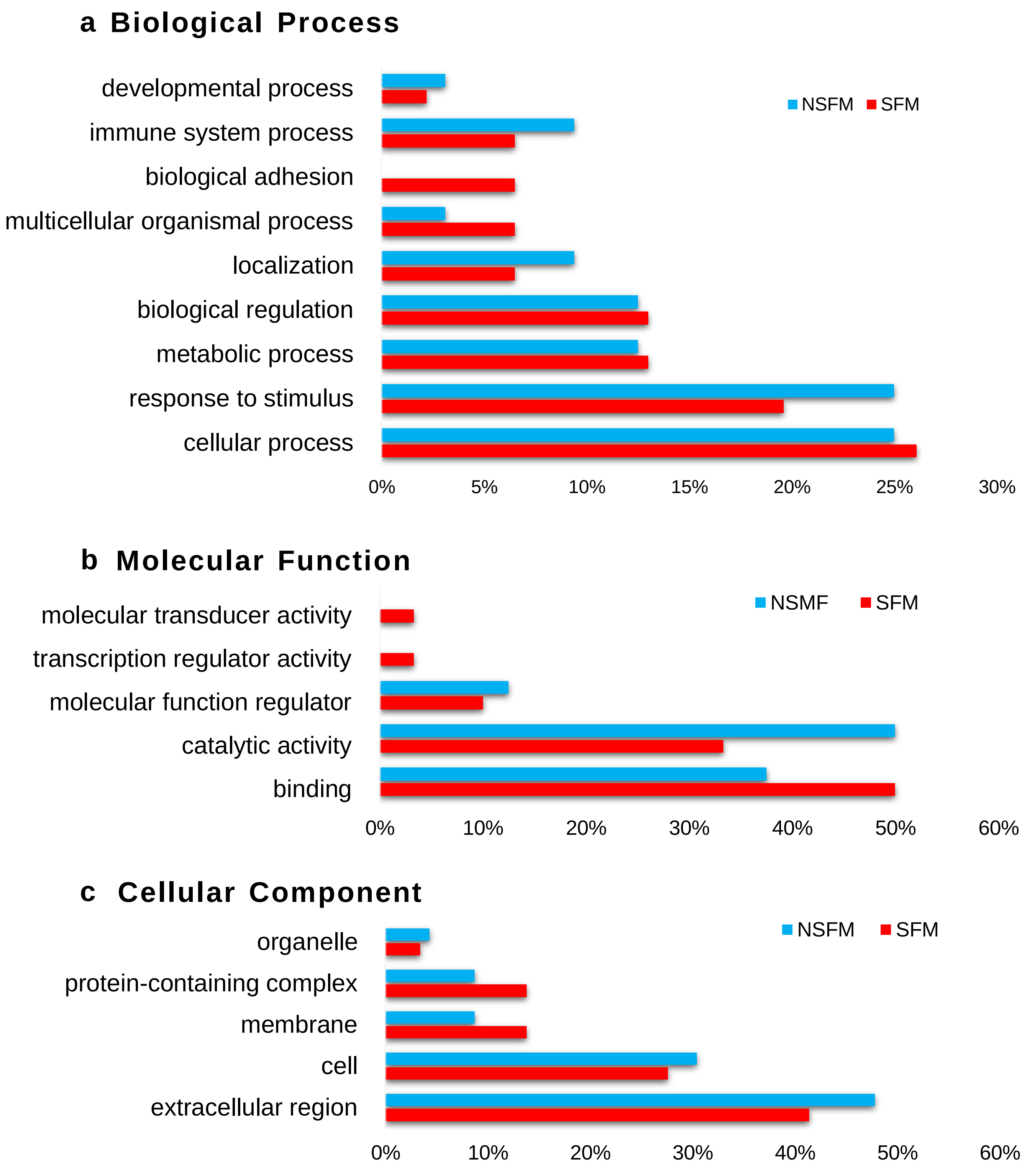

Supplementary Fig. 6 Gene ontology (GO) enrichment analysis of the differentially abundant plasma proteins identified in falciparum malaria patients: **a-c** Distribution of GO terms exhibiting functional classification in non-severe falciparum malaria and severe falciparum malaria. Biological Process **a**, Molecular Function **b**, Cellular Component **c**.

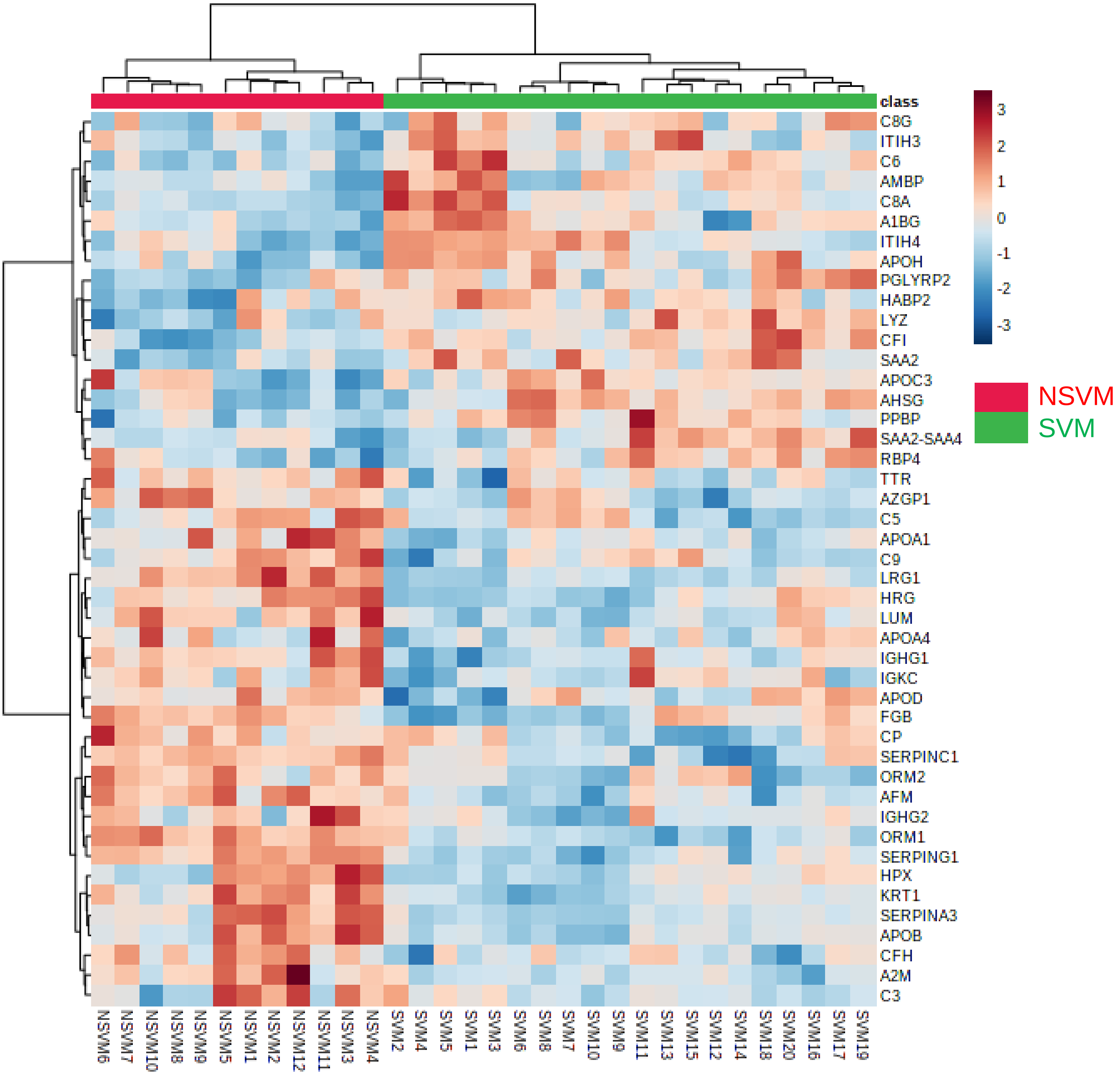

Supplementary Fig. 7 Unsupervised clustering of differentially abundant proteins ($p < 0.05$) in vivax malaria. Heat map of individual samples for non-severe vivax malaria (NSVM) and severe vivax malaria (SVM)

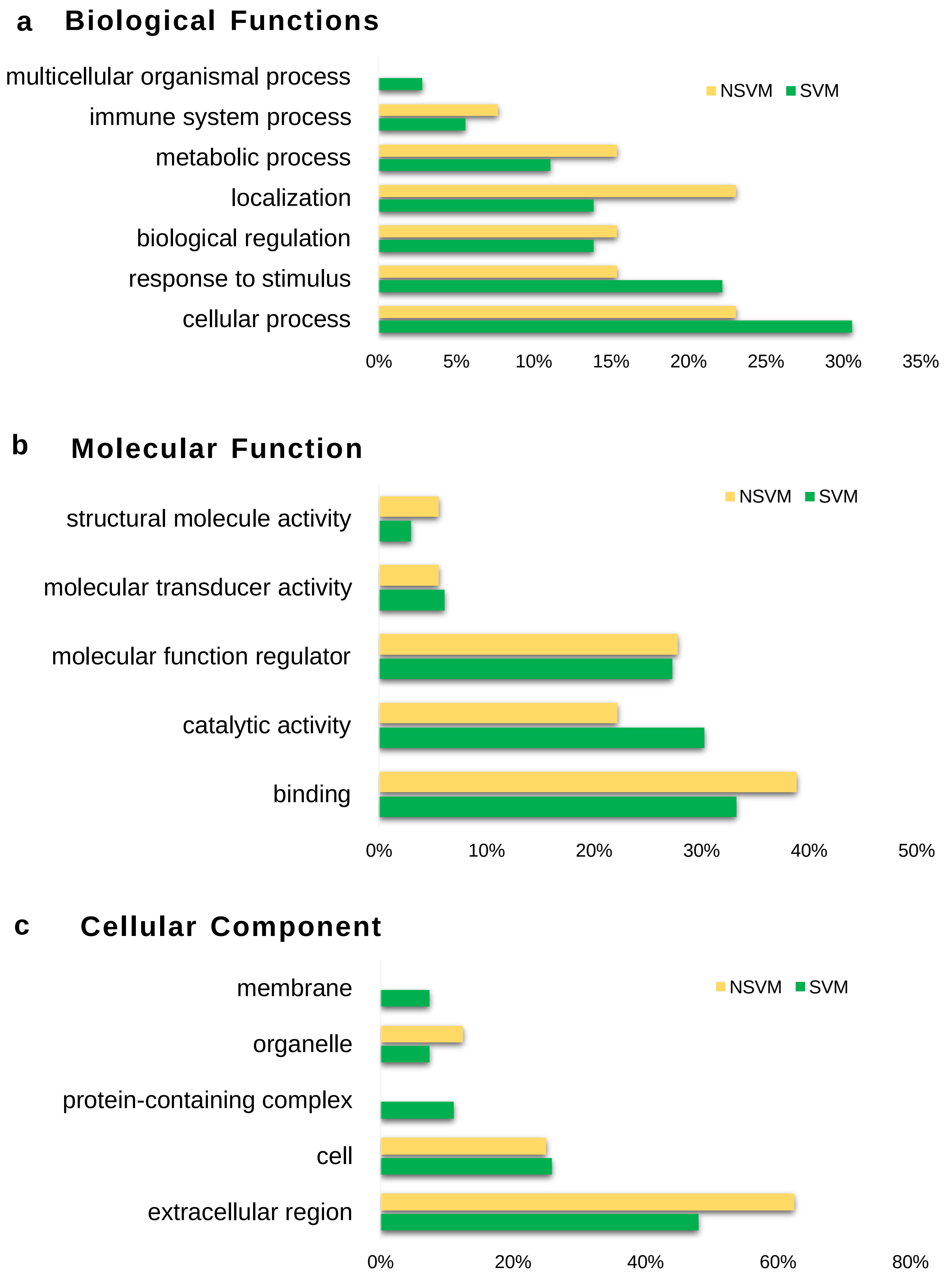

Supplementary Fig. 8 Gene ontology (GO) enrichment analysis of the differentially abundant plasma proteins identified in vivax malaria patients. **a-c** Distribution of GO terms exhibiting functional classification in non-severe vivax malaria (NSVM) and severe vivax malaria (SVM). Biological Process **a**, Molecular Function **b**, Cellular Component **c**.

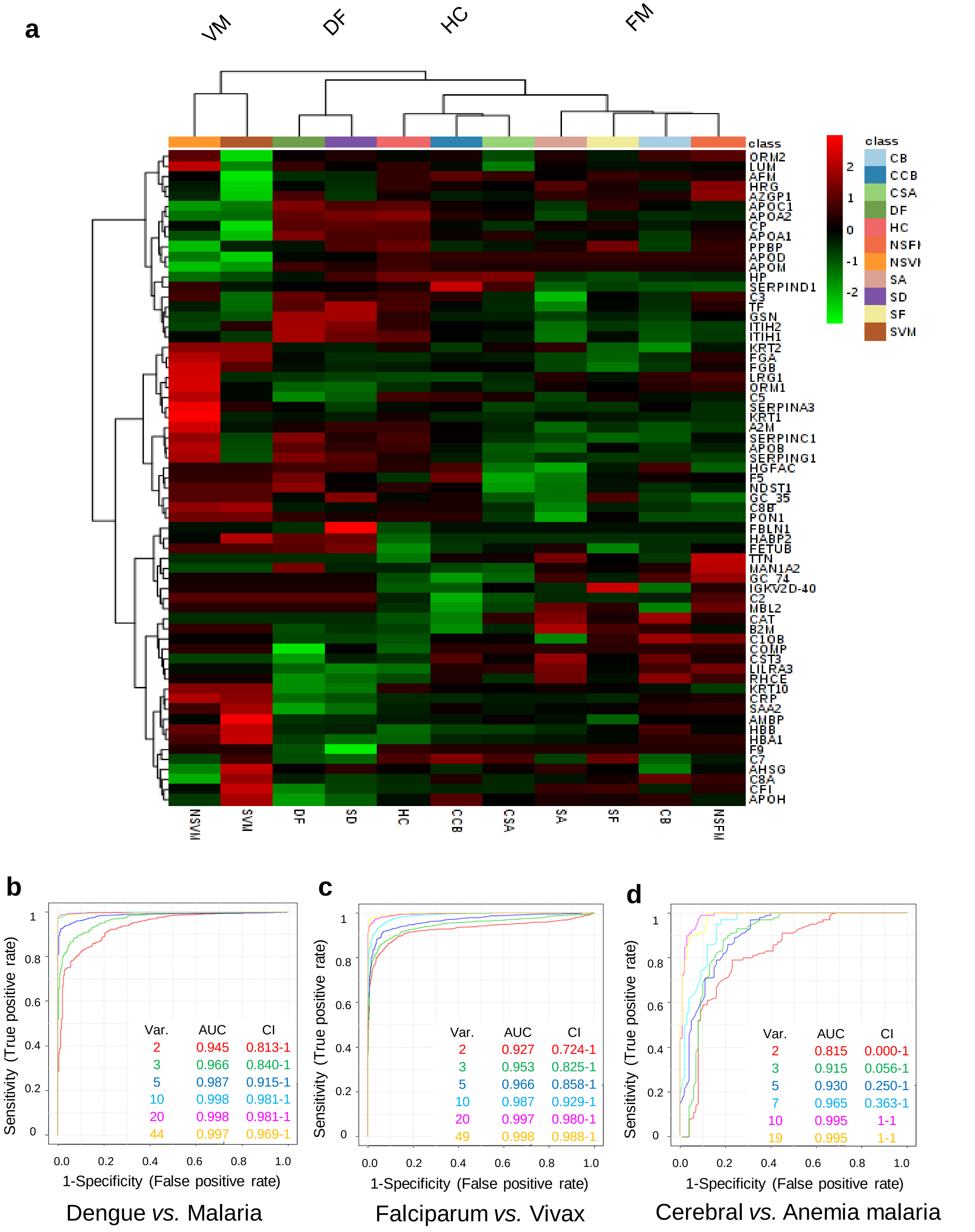

Supplementary Fig. 9 Machine learning model to identify biomarker signatures. **a** Heat map of input list of significant altered proteins for machine learning. **b-d** ROC curve of best panel of protein biomarker. Dengue *vs*. Malaria **b**. Falciparum *vs*. Vivax **c**, Cerebral *vs*. anemia malaria **d**.

a

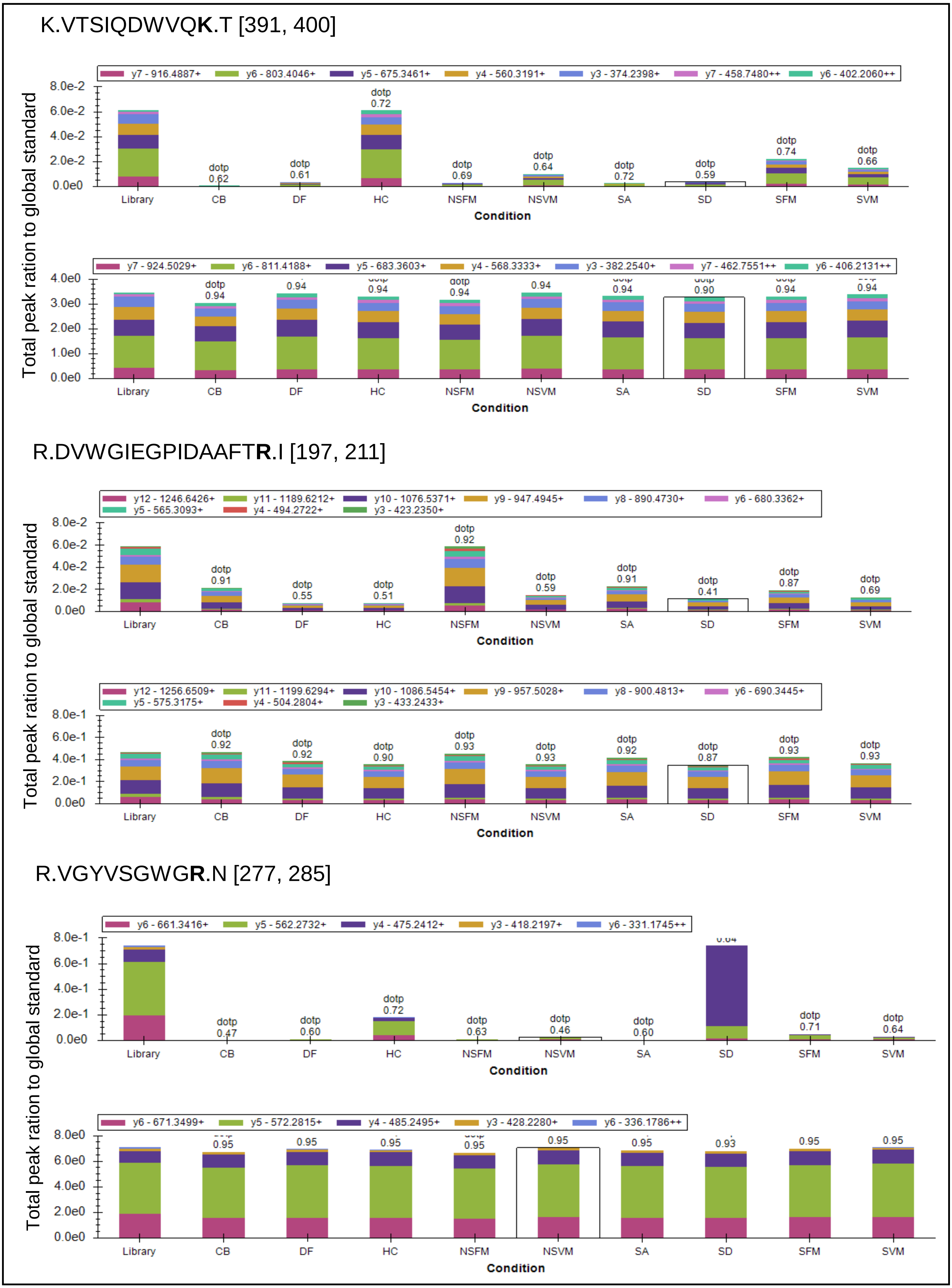
K.VTSIQDWVQ**K**.T [391, 400]
y7 - 916.4887+ y6 - 803.4046+ y5 - 675.3461+ y4 - 560.3191+ y3 - 374.2398+ y7 - 458.7480++ y6 - 402.2060++
8.0e-2 6.0e-2 4.0e-2 2.0e-2 0.0e0
dotp 0.62 dotp 0.61 dotp 0.72 dotp 0.69 dotp 0.64 dotp 0.72 dotp 0.59 dotp 0.74 dotp 0.66
Library CB DF HC NSFM NSVM SA SD SFM SVM
Condition
y7 - 924.5029+ y6 - 811.4188+ y5 - 683.3603+ y4 - 568.3333+ y3 - 382.2540+ y7 - 462.7551++ y6 - 406.2131++
4.0e0 3.0e0 2.0e0 1.0e0 0.0e0
0.94 0.94 0.94 0.94 0.94 0.94 0.90 0.94 0.94
Library CB DF HC NSFM NSVM SA SD SFM SVM
Condition
Total peak ration to global standard
R.DVWGIEGPIDAAFT**R**.I [197, 211]
y12 - 1246.6426+ y11 - 1189.6212+ y10 - 1076.5371+ y9 - 947.4945+ y8 - 890.4730+ y6 - 680.3362+ y5 - 565.3093+ y4 - 494.2722+ y3 - 423.2350+
8.0e-2 6.0e-2 4.0e-2 2.0e-2 0.0e0
dotp 0.91 dotp 0.55 dotp 0.51 dotp 0.92 dotp 0.59 dotp 0.91 dotp 0.41 dotp 0.87 dotp 0.69
Library CB DF HC NSFM NSVM SA SD SFM SVM
Condition
y12 - 1256.6509+ y11 - 1199.6294+ y10 - 1086.5454+ y9 - 957.5028+ y8 - 900.4813+ y6 - 690.3445+ y5 - 575.3175+ y4 - 504.2804+ y3 - 433.2433+
8.0e-1 6.0e-1 4.0e-1 2.0e-1 0.0e0
dotp 0.92 dotp 0.92 dotp 0.90 dotp 0.93 dotp 0.93 dotp 0.92 dotp 0.87 dotp 0.93 dotp 0.93
Library CB DF HC NSFM NSVM SA SD SFM SVM
Condition
Total peak ration to global standard
R.VGYVSGWG**R**.N [277, 285]
y6 - 661.3416+ y5 - 562.2732+ y4 - 475.2412+ y3 - 418.2197+ y6 - 331.1745++
8.0e-1 6.0e-1 4.0e-1 2.0e-1 0.0e0
dotp 0.47 dotp 0.60 dotp 0.72 dotp 0.63 dotp 0.46 dotp 0.60 dotp 0.71 dotp 0.64
Library CB DF HC NSFM NSVM SA SD SFM SVM
Condition
y6 - 671.3499+ y5 - 572.2815+ y4 - 485.2495+ y3 - 428.2280+ y6 - 336.1786++
8.0e0 6.0e0 4.0e0 2.0e0 0.0e0
0.95 0.95 0.95 0.95 0.95 0.95 0.93 0.95 0.95
Library CB DF HC NSFM NSVM SA SD SFM SVM
Condition
Total peak ration to global standard

Contd.

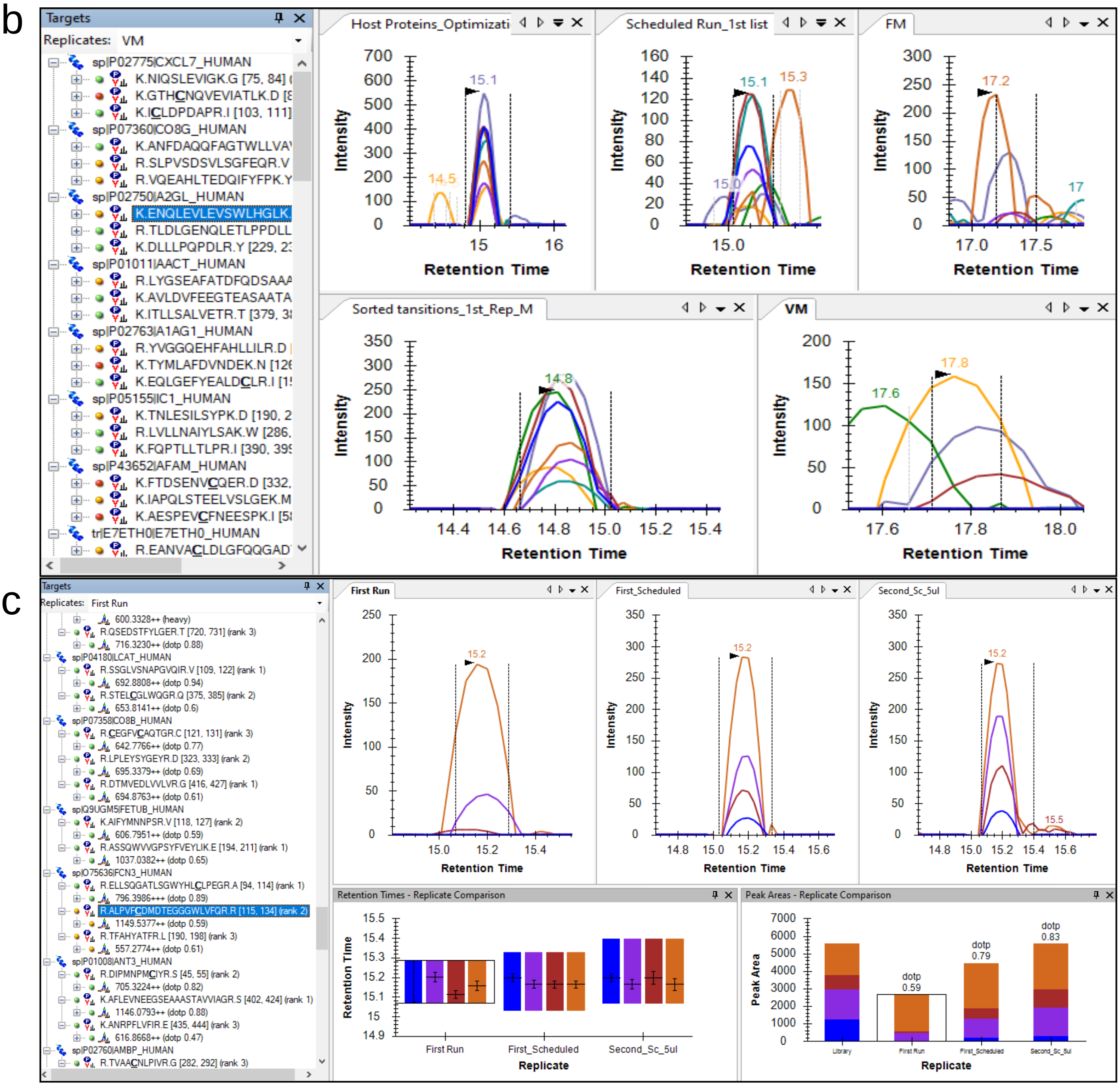

Supplementary Fig. 10 Optimization of Transition for MRM experiment: a The peak area ratio to global standard was shown of some representative heavy synthetic peptides to check the system suitability. **b** The representative screenshot of the Skyline interface, which allows to select the best peptide by monitoring the peak shape. The peptides, which has distorted peak shape are deleted from the list of the peptides**. c** The transition of same peptide is optimized by scheduling the run. The dotp value shows the similarity between the obtained MS spectra with existing spectral library. The scale of dotp is between 0 and 1. So the values closer to 1, indicate more similarity with spectral library, and hence more significant.

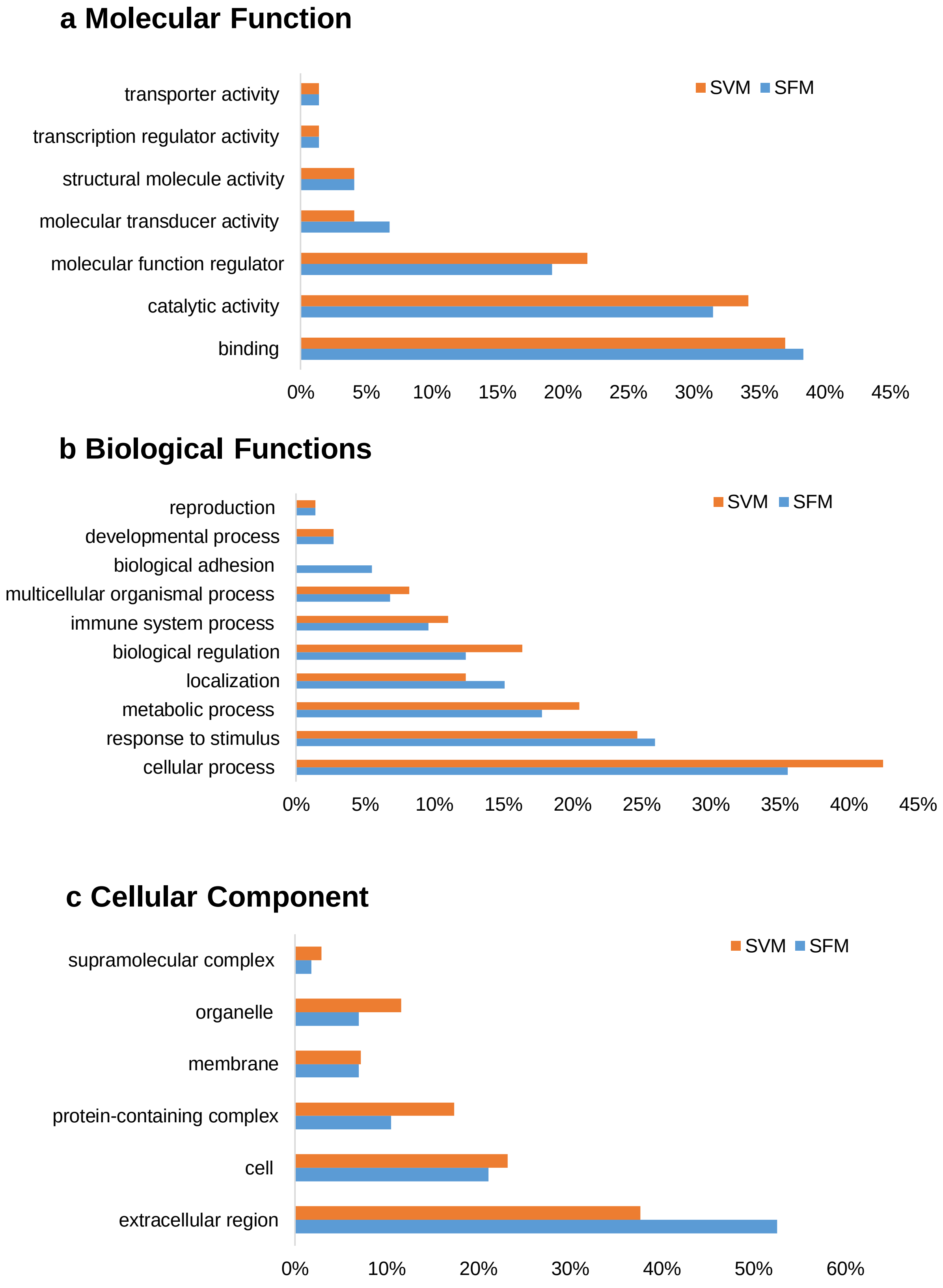

Supplementary Fig. 11 Gene ontology (GO) enrichment analysis of the differentially abundant plasma proteins identified severe falciparum and severe vivax malaria patients. **a-c** Distribution of GO terms exhibiting functional classification in severe falciparum malaria (SFM) and severe vivax malaria (SVM). Biological Process **a**, Molecular Function **b**, Cellular Component **c**.

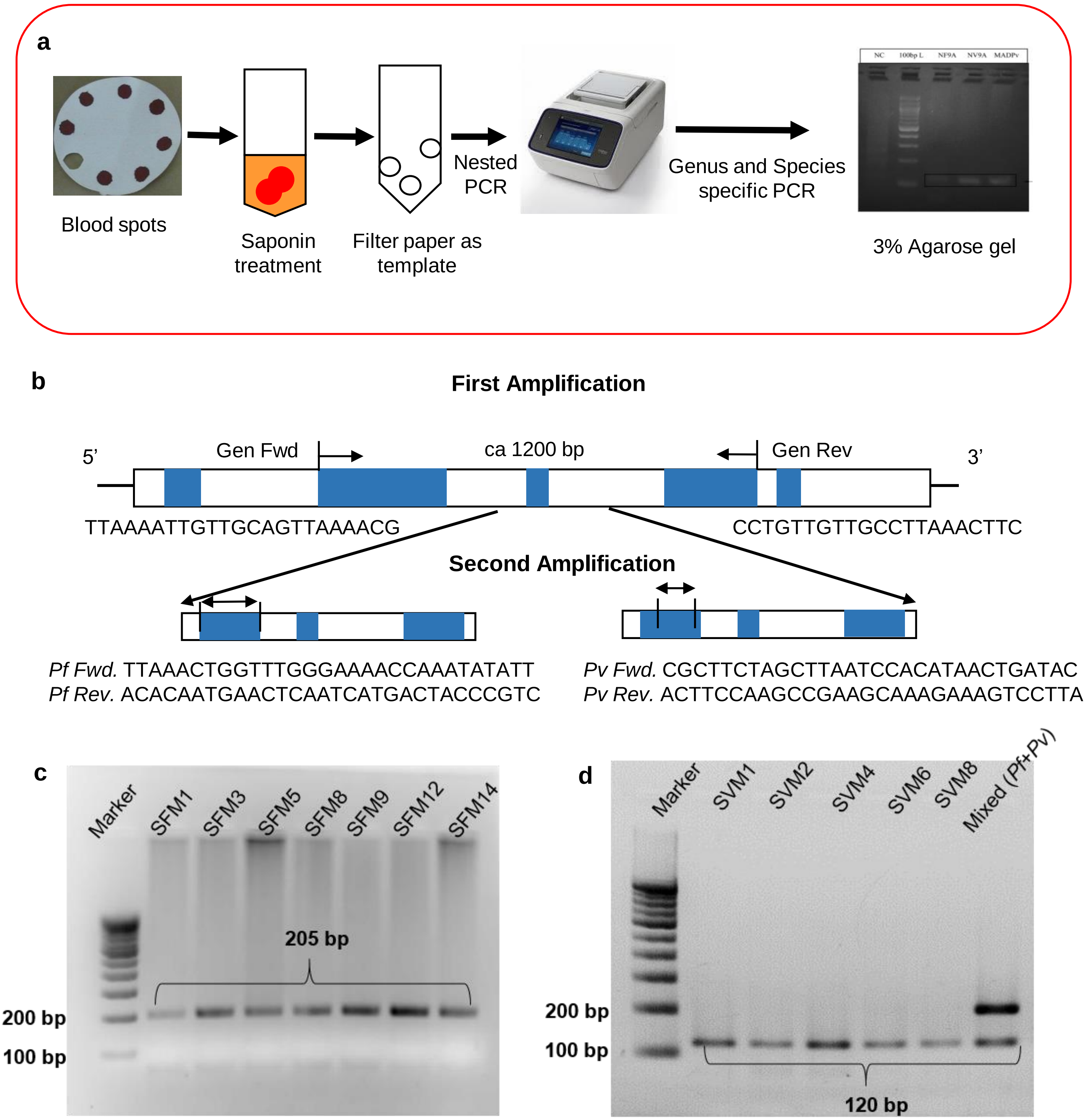

Supplementary Fig. 12 PCR confirmation for diagnosis of malaria patients. **a** Workflow of nested PCR using dried blood spots as the template for the reaction. **b** Primers for nested PCR for various species of *Plasmodium falciparum* and Plasmodium vivax confirmation from malaria infected blood samples. **c-d** samples of falciparum **c** and vivax **d** infected patients, band at 120bp represents vivax infection whereas band at 205 bp represents falciparum infection.

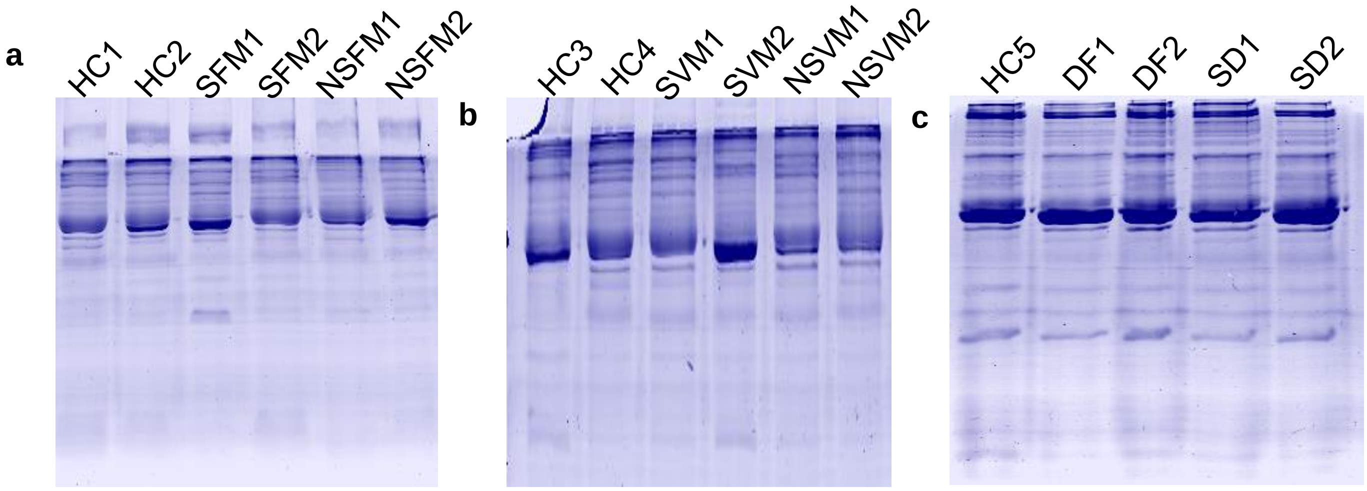

d

TMT	Reaction 1	Reaction 2	Reaction 3	Reaction 4	Reaction 5	Reaction 6	Reaction 7	Reaction 8	Reaction 9	Reaction 10
126	Pool1	Pool2	Pool3	Pool4	Pool5	Pool6	Pool7	Pool8	Pool9	Pool10
127	HC1	HC2	HC3	HC4	HC5	HC6	HC7	HC8	HC9	HC10
128	NSFM1	NSFM2	NSFM3	NSFM4	NSFM5	NSFM6	NSFM7	NSFM8	SF1	SF2
129	CSA1	CSA2	CSA3	CCB1	CCB2	CCB3	CSA4	CCB4	SF3	SF4
130	SA1	SA2	SA3	CB1	CB2	CB3	SF5	SF6	SF7	SF8
131	SA4	SA5	SA6	CB4	CB5	CB6	SF9	SF10	SF11	SF12

e

TMT	Reaction 1	Reaction 2	Reaction 3	Reaction 4
126	Pool1	Pool2	Pool3	Pool4
127N	HC1	HC2	HC3	HC4
127C	NSVM1	NSVM4	NSVM7	NSVM10
128N	NSVM2	NSVM5	NSVM8	NSVM11
128C	NSVM3	NSVM6	NSVM9	NSVM12
129N	SVM1	SVM6	SVM11	SVM16
129C	SVM2	SVM7	SVM12	SVM17
130N	SVM3	SVM8	SVM13	SVM18
130C	SVM4	SVM9	SVM14	SVM19
131	SVM5	SVM10	SVM15	SVM20

f

TMT	Reaction 1	Reaction 2	Reaction 3	Reaction 4
126	Pool1	Pool2	Pool3	Pool4
127	HC1	DF2	SD7	SD10
128	SD1	HC2	DF3	SD11
129	DF1	SD4	HC3	SD12
130	SD1	SD5	SD8	HC4
131	SD1	SD6	SD9	DF4

Supplementary Fig. 13 Quality control check of depleted plasma samples and the labeling strategy used in TMT-based quantitative proteomics analysis. **a-c** some representative SDS gel images for falciparum malaria plasma samples **a**, vivax malaria **b** and Dengue samples **c**. **d** The experimental plan for falciparum malaria quantitative proteomics analysis using TMT6-plex reagents. **e** The experiment plan for TMT labeling of vivax malaria samples using TMT10-plex reagents. **f** The experimental plan used to label the dengue samples using TMT6-plex reagents.
