# Supplementary material for: Multiplexed quantitative proteomics provides mechanistic cues for malaria severity and complexity
Source: Commun Biol. 2020 Nov 17;3:683. doi: 10.1038/s42003-020-01384-4 (PMC7672109; doi:10.1038/s42003-020-01384-4)
Supplement: Supplementary file 3 — Description of Additional Supplementary Files [file 42003_2020_1384_MOESM3_ESM.pdf]

Description of Additional Supplementary Files

File Name: Supplementary Data 1
Description: Details of sample used in discovery phase quantitative proteomics, targeted validation, and clinicopathological analysis.

File Name: Supplementary Data 2
Description: Human altered proteins in plasma of falciparum patients as compared to healthy individuals.

File Name: Supplementary Data 3
Description: Human altered proteins in plasma of vivax infected patients as compared to healthy individuals.

File Name: Supplementary Data 4
Description: The list of the parasite proteins identified from plasma samples of severe falciparum malaria.

File Name: Supplementary Data 5
Description: Prediction of best possible candidates to distinguish various febrile conditions using a machine learning approach.

File Name: Supplementary Data 6
Description: Lists of peptides for validation study using MS-based Multiple Reaction Monitoring (MRM) assays.

File Name: Supplementary Data 7
Description: Human altered proteins in plasma of dengue infected patient as compared to healthy individuals.
