# Supplementary material for: Multiplexed quantitative proteomics provides mechanistic cues for malaria severity and complexity
Source: Commun Biol. 2020 Nov 17;3:683. doi: 10.1038/s42003-020-01384-4 (PMC7672109; doi:10.1038/s42003-020-01384-4)
Supplement: Supplementary file 11 — Reporting Summary [file 42003_2020_1384_MOESM11_ESM.pdf]

nature research

Corresponding author(s): Prof. Sanjeeva Srivastava, COMMSBIO-20-0327B

Last updated by author(s): Oct 5, 2020

# Reporting Summary

Nature Research wishes to improve the reproducibility of the work that we publish. This form provides structure for consistency and transparency in reporting. For further information on Nature Research policies, see our Editorial Policies and the Editorial Policy Checklist.

## Statistics

For all statistical analyses, confirm that the following items are present in the figure legend, table legend, main text, or Methods section.

| n/a | Confirmed | |
|---|---|---|
| ☐ | ☒ | The exact sample size ($n$) for each experimental group/condition, given as a discrete number and unit of measurement |
| ☐ | ☒ | A statement on whether measurements were taken from distinct samples or whether the same sample was measured repeatedly |
| ☐ | ☒ | The statistical test(s) used AND whether they are one- or two-sided<br>*Only common tests should be described solely by name; describe more complex techniques in the Methods section.* |
| ☒ | ☐ | A description of all covariates tested |
| ☐ | ☒ | A description of any assumptions or corrections, such as tests of normality and adjustment for multiple comparisons |
| ☐ | ☒ | A full description of the statistical parameters including central tendency (e.g. means) or other basic estimates (e.g. regression coefficient) AND variation (e.g. standard deviation) or associated estimates of uncertainty (e.g. confidence intervals) |
| ☐ | ☒ | For null hypothesis testing, the test statistic (e.g. $F$, $t$, $r$) with confidence intervals, effect sizes, degrees of freedom and $P$ value noted<br>*Give P values as exact values whenever suitable.* |
| ☒ | ☐ | For Bayesian analysis, information on the choice of priors and Markov chain Monte Carlo settings |
| ☐ | ☒ | For hierarchical and complex designs, identification of the appropriate level for tests and full reporting of outcomes |
| ☒ | ☐ | Estimates of effect sizes (e.g. Cohen's $d$, Pearson's $r$), indicating how they were calculated |

*Our web collection on statistics for biologists contains articles on many of the points above.*

## Software and code

Policy information about availability of computer code

| | |
|---|---|
| Data collection | Quantitative proteomics data were extracted from the raw files using Thermo Proteome Discoverer 2.2.0.388 |
| Data analysis | Data were analyzed in R (3.5.1) using the MSstatsTMT Package (0.99.6). The custom code for MS data analysis (TMT6/10plex) and machine learning have been deposited at https://github.com/vipin786/R-code-for-MS-data as well as publicly available via Zenodo (http://doi.org/10.5281/zenodo.4022347). |

For manuscripts utilizing custom algorithms or software that are central to the research but not yet described in published literature, software must be made available to editors and reviewers. We strongly encourage code deposition in a community repository (e.g. GitHub). See the Nature Research guidelines for submitting code & software for further information.

## Data

Policy information about availability of data

All manuscripts must include a data availability statement. This statement should provide the following information, where applicable:

- Accession codes, unique identifiers, or web links for publicly available datasets
- A list of figures that have associated raw data
- A description of any restrictions on data availability

All processed data associated with this study are present in the manuscript or in the Supplementary Materials. Raw MS data and search output files for TMT-based quantitative proteomics analyses described in this article are deposited to the ProteomeXchange Consortium via the PRIDE partner repository with the dataset identifier PXD014991. Targeted proteomic data are deposited in the Peptide Atlas and can be accessed through Dataset Identifier: PASS01467. All raw and processed data are made available as supplementary datasets 1-7.



April 2020

# Field-specific reporting

Please select the one below that is the best fit for your research. If you are not sure, read the appropriate sections before making your selection.

[x] Life sciences  [ ] Behavioural & social sciences  [ ] Ecological, evolutionary & environmental sciences

For a reference copy of the document with all sections, see nature.com/documents/nr-reporting-summary-flat.pdf

# Life sciences study design

All studies must disclose on these points even when the disclosure is negative.

| | |
|---|---|
| Sample size | We included different number of subjects (n >>3) in different comparative experiments. The minimum number of subjects per group was determined following our earlier studies. We have provided the details of each sample in supplementary Table 1. |
| Data exclusions | n/a |
| Replication | Multiple biological replicates for each condition were analyzed and further technical replicates were included wherever required. |
| Randomization | Samples were randomized in the validation phase (Targeted proteomics analysis) |
| Blinding | n/a |

# Reporting for specific materials, systems and methods

We require information from authors about some types of materials, experimental systems and methods used in many studies. Here, indicate whether each material, system or method listed is relevant to your study. If you are not sure if a list item applies to your research, read the appropriate section before selecting a response.

## Materials & experimental systems

| n/a | Involved in the study | |
|---|---|---|
| [x] | [ ] | Antibodies |
| [x] | [ ] | Eukaryotic cell lines |
| [x] | [ ] | Palaeontology and archaeology |
| [x] | [ ] | Animals and other organisms |
| [ ] | [x] | Human research participants |
| [x] | [ ] | Clinical data |
| [x] | [ ] | Dual use research of concern |

## Methods

| n/a | Involved in the study | |
|---|---|---|
| [x] | [ ] | ChIP-seq |
| [x] | [ ] | Flow cytometry |
| [x] | [ ] | MRI-based neuroimaging |

## Human research participants

Policy information about studies involving human research participants

| | |
|---|---|
| Population characteristics | This study was conducted involving malaria and dengue fever patients, and control subjects enrolled in the CMC medical college, Kolkata, India, and the SPMC medical college, Bikaner, India. |
| Recruitment | The clinicopathological details of all the subjects enrolled in this study were thoroughly documented. The diagnosis of malaria positive samples was carried out primarily by microscopic examination of thin peripheral blood smears followed by rapid diagnostic tests (RDTs, FalciVax, Zephyr Biomedicals). |
| Ethics oversight | This study was approved by the institutional review boards and ethics committee of the Indian Institute of Technology Bombay (IITB-IEC/2016/026). |

Note that full information on the approval of the study protocol must also be provided in the manuscript.
